# Presepsin (soluble CD14 subtype) and procalcitonin levels for mortality prediction in sepsis: data from the Albumin Italian Outcome Sepsis trial

**DOI:** 10.1186/cc13183

**Published:** 2014-01-07

**Authors:** Serge Masson, Pietro Caironi, Eberhard Spanuth, Ralf Thomae, Mauro Panigada, Gabriela Sangiorgi, Roberto Fumagalli, Tommaso Mauri, Stefano Isgrò, Caterina Fanizza, Marilena Romero, Gianni Tognoni, Roberto Latini, Luciano Gattinoni

**Affiliations:** 1 IRCCS - Istituto di Ricerche Farmacologiche “Mario Negri”, via Giuseppe La Masa 19, 20156 Milan, Italy; 2 Dipartimento di Fisiopatologia Medico-Chirurgica e dei Trapianti, Fondazione IRCCS Ca' Granda - Ospedale Maggiore Policlinico, Università degli Studi di Milano, via Francesco Sforza 35, 20122 Milan, Italy; 3 Dipartimento di Anestesia, Rianimazione, e Terapia del Dolore, Fondazione IRCCS Ca’ Granda – Ospedale Maggiore Policlinico, via Francesco Sforza 35, 20122 Milan, Italy; 4 Diagnostics Engineering & Research GmbH, Friedrichstrasse 26, 69221 Heidelberg, Germany; 5 Mitsubishi Chemical Europe GmbH, Willstätterstrasse 30, 40549 Düsseldorf, Germany; 6 Anestesiologia e Rianimazione, Dipartimento Emergenza/Urgenza, Chirurgia Generale e dei Trapianti; Policlinico Universitario S. Orsola Malpighi, via Pietro Albertoni, 15, 40138 Bologna, Italy; 7 UO Anestesia e Rianimazione, AO San Gerardo, via Giambattista Pergolesi, 33, 20900 Monza, Italy; 8 Dipartimento di Emergenza, Ospedale San Gerardo and Milano-Bicocca University, via Giambattista Pergolesi, 33, 20900 Monza, Italy; 9 Consorzio Mario Negri Sud, via Nazionale 8/A, 66030 Santa Maria Imbaro, Italy

## Abstract

**Introduction:**

Sepsis, a leading cause of death in critically ill patients, is the result of complex interactions between the infecting microorganisms and the host responses that influence clinical outcomes. We evaluated the prognostic value of presepsin (sCD14-ST), a novel biomarker of bacterial infection, and compared it with procalcitonin (PCT).

**Methods:**

This is a retrospective, case–control study of a multicenter, randomized clinical trial enrolling patients with severe sepsis or septic shock in ICUs in Italy. We selected 50 survivors and 50 non-survivors at ICU discharge, matched for age, sex and time from sepsis diagnosis to enrollment. Plasma samples were collected 1, 2 and 7 days after enrollment to assay presepsin and PCT. Outcome was assessed 28 and 90 days after enrollment.

**Results:**

Early presepsin (day 1) was higher in decedents (2,269 pg/ml, median (Q1 to Q3), 1,171 to 4,300 pg/ml) than in survivors (1,184 pg/ml (median, 875 to 2,113); *P* = 0.002), whereas PCT was not different (18.5 μg/L (median 3.4 to 45.2) and 10.8 μg/L (2.7 to 41.9); *P* = 0.31). The evolution of presepsin levels over time was significantly different in survivors compared to decedents (*P* for time-survival interaction = 0.03), whereas PCT decreased similarly in the two groups (*P* = 0.13). Presepsin was the only variable independently associated with ICU and 28-day mortality in Cox models adjusted for clinical characteristics. It showed better prognostic accuracy than PCT in the range of Sequential Organ Failure Assessment score (area under the curve (AUC) from 0.64 to 0.75 vs. AUC 0.53 to 0.65).

**Conclusions:**

In this multicenter clinical trial, we provide the first evidence that presepsin measurements may have useful prognostic information for patients with severe sepsis or septic shock. These preliminary findings suggest that presepsin may be of clinical importance for early risk stratification.

## Introduction

Sepsis is the leading cause of death in critically ill patients and requires early goal-directed management to reduce its high burden of mortality and morbidity [1]. During sepsis, the combination of severe infection and the subsequent nonlocalized inflammatory and immune-mediated systemic responses may result in a clinical condition with lethality as high as 40% [2]. Despite efforts to improve early recognition and clinical treatment, sepsis often evolves to septic shock (that is, reduced tissue perfusion despite fluid therapy and vasoactive drugs) and multiorgan failure, which are the most frequent causes of death in the septic patient. The host’s innate and adaptive immune responses are fundamental in defense against the infecting microorganisms, but they also contribute to the amplification of proinflammatory mechanisms, coagulation imbalance and endothelial dysfunction that participate in organ injury [3].

Optimal management requires early goal-oriented therapies, so, in principle, it could be improved by individualized circulating biomarkers for early risk stratification. Circulating biomarkers may help in making the diagnosis and guiding antimicrobial therapy for patients with sepsis [4,5], but few have proved to be useful for individual prognostic stratification. Presepsin (sCD14-ST) is a soluble N-terminal fragment of the cluster of differentiation (CD) marker protein CD14, which is released into the circulation during monocyte activation upon the recognition of lipopolysaccharide (LPS) from infectious agents [6]. It shows promise for diagnostic purposes [7] and powerful prognostic information in septic patients as early as the time of admission [8]. To date, no multicenter study has been conducted to evaluate the prognostic value of presepsin during severe sepsis. We therefore set out to examine the relationships between early plasma presepsin concentration and mortality in patients with severe sepsis and septic shock and compare its prognostic performance with that of procalcitonin (PCT).

## Materials and methods

### Study design

This retrospective case–control study was conducted with data from the multicenter, randomized Albumin Italian Outcome Sepsis (ALBIOS) trial, which enrolled patients with severe sepsis or septic shock from 100 ICUs in Italy (Clinicaltrials.gov Identifier: NCT00707122). The primary aim of the trial was to verify whether volume replacement with albumin and the maintenance of serum albumin levels within the physiologic range during the first 28 days (or until ICU discharge, whichever came first) improved 28-day and 90-day survival as compared to crystalloids. Both arms of the study were carried out in accordance with the Surviving Sepsis Campaign guidelines for early goal-directed therapy and the treatment of patients with severe sepsis [1,9].

We selected 50 survivors and 50 nonsurvivors at the time of ICU discharge (21 ± 18 days). The cohorts were matched for age, sex, source center and time of enrollment after confirmation of the inclusion criteria (within 6 hours and between 6 and 24 hours), which were at least one focus of infection (known or suspected), two or more signs of systemic inflammatory reaction syndrome (core temperature >38°C or <36°C; heart rate >90 beats/min; respiratory rate >20 breaths/min or arterial partial pressure of carbon dioxide <32 mmHg or requirement for mechanical ventilation for an acute pathological process; white blood cell count >12,000/μl or <4,000/μl or more than 10% immature neutrophils) and at least one severe, acute sepsis-related organ dysfunction assessed on the basis of the Sequential Organ Failure Assessment (SOFA) score [10]. Exclusion criteria were age younger than 18 years, terminal state, a known adverse reaction to albumin, a proved or suspected and clinically active brain injury, congestive heart failure (New York Heart Association class III or IV), a pathological condition in which albumin could be clinically indicated (such as cirrhosis with ascites, intestinal malabsorption syndrome, nephrotic syndrome or burns), more than 24 hours elapsed since the presence of inclusion criteria, patient’s religious objection to the administration of human blood products and inclusion in other experimental studies. Assignment to the randomization arm was not known when patients were selected for the present case-control study.

Clinical data on hemodynamic parameters, vasoactive drug administration, blood gas analysis, ventilatory status, fluid balance, antibiotic therapy and standard laboratory tests were obtained daily from the day of study enrollment to day 28 (or until ICU discharge, whichever came first). The Simplified Acute Physiology Score II (SAPS II) was recorded within the first 24 hours after enrollment, and the SOFA score was calculated daily. For consistency between the indications provided by the current guidelines for the management of severe sepsis and septic shock [1] and for the calculation of the SOFA score [10], the cardiovascular SOFA subscore was modified by lowering the mean arterial pressure limit to 65 mmHg for point 1. Moreover, the aggregate SOFA score did not include neurological function, as cerebral failure was not assessed during the study [11-13]. Data on the initial site of infection and the infecting microorganisms (based on infection site and blood culture) were collected periodically. Septic shock at the time of randomization was defined as a cardiovascular component of the SOFA score of 1, 3 or 4 [11].

A complete list of centers and investigators participating in the ALBIOS substudy on biomarkers is presented in Additional file 1. This study was compliant with the 1975 Declaration of Helsinki as revised in 2008 and was approved first by the Institutional Review Board of the Fondazione IRCCS Ca' Granda - Ospedale Maggiore Policlinico, Milan, Italy (coordinating center), and subsequently by the appropriate institutional review boards of all the other participating centers (see Additional file 2). Written informed consent or deferred consent was obtained from all participants.

### Sample collection and circulating biomarker measurement

Ethylenediaminetetraacetic acid plasma samples were serially collected 1, 2 and 7 days after enrollment (or at ICU discharge, whichever came first), shipped on dry ice to a central repository and stored at -70°C until assayed. A central laboratory whose technicians were blinded to patients’ characteristics measured presepsin (chemiluminescent enzyme immunoassay, PATHFAST Presepsin, Mitsubishi Chemical) [14], and procalcitonin (Elecsys B.R.A.H.M.S Cobas^®^ PCT, Roche Diagnostics). For imprecision determination, four plasma samples were assayed in duplicate on 20 nonconsecutive days. The PATHFAST Presepsin assay revealed intra-assay and interassay coefficients of variation (CVs) of 4.4% at 445 pg/ml. At 4,901 pg/ml, intra- and interassay CVs of 3.2% and 3.9% were obtained, respectively. The limit of detection was 20 pg/ml. The Elecsys BRAHMS Cobas^®^ PCT assay yielded a limit of detection of 0.02 μg/L. The intra-assay and interassay CVs were 9.9% and 16.3% at a PCT concentration of 0.06 μg/L and 2.1% and 4.2% at 41.2 μg/L, respectively. Reference values (95th percentile) measured in a control group of 110 healthy volunteers were 320 pg/ml for presepsin (upper limit of normal) and 0.046 μg/L for PCT.

### Outcomes and statistical analysis

The primary outcome measure of the ALBIOS trial was death recorded 28 and 90 days after enrollment. We also evaluated the association between circulating biomarkers and survival at ICU discharge. Categorical variables are presented as proportions and continuous variables as means (±SD) or medians (Q1 to Q3). Differences in clinical characteristics according to survival status were analyzed using the χ^2^ test or Fisher’s exact test for categorical variables. Continuous variables were compared by performing a two-sample *t*-test or the nonparametric Mann–Whitney *U* test for non-normally distributed data. Changes of biomarker concentrations over time in survivors and decedents were compared using two-way analysis of variance for repeated measurements on log-transformed data when appropriate. The comparison of presepsin and PCT concentrations at 1, 2 and 7 days between decedents and survivors at ICU discharge was performed using the Mann–Whitney *U* test in both the overall study population and the subgroup of patients with septic shock.

The relation between circulating biomarker concentrations, entered as log-transformed continuous variables, and mortality was first assessed with univariate Cox proportional hazards models, and these data are presented as hazard ratios (HRs) and 95% confidence intervals (CIs) for a 1-unit increase on a logarithmic scale. Cumulative sums of martingale-based residuals, plots and testing were used to evaluate whether an independent covariate could be entered directly into the model or if a transformation was necessary. The effect of the absence of the time dependence on the ability to predict outcomes was confirmed by using the calculation method proposed by Lin and colleagues [15], derived from the same analysis of cumulative sums of martingale-based residuals.

Cox multivariate models were used to establish the independent prognostic value of circulating biomarkers after adjustment for a set of clinically relevant variables set *a priori*: SAPS II score; SOFA score and serum lactate concentration, both measured on day 1; mean arterial pressure and central venous oxygen saturation, both measured at 6 hours after enrollment; and randomized treatment (albumin vs. crystalloids). All of these covariates were directly included in the model. The prognostic discrimination of each biomarker and two clinical risk scores (SOFA and SAPS II scores) was established on the basis of the area under the receiver operating characteristic (ROC) curve, which is equivalent to the C-index. The optimal cutoff was chosen as the highest product of sensitivity and specificity. Statistical analysis was done using SAS version 9.2 software (SAS Institute, Cary, NC, USA). A two-sided *P* value <0.05 was deemed to be statistically significant.

## Results

### Presepsin concentration and baseline clinical characteristics

Additional file 3 gives the selected clinical characteristics of patients at the time of study enrollment according to median levels of presepsin assessed on day 1 (1,494 pg/ml). Higher levels of presepsin were significantly associated with worse SOFA scores and reduced diuresis.

### Time course of presepsin levels during the study period

Baseline clinical characteristics and circulating biomarker concentrations measured at day 1 in survivors and nonsurvivors at ICU discharge are presented in Table 1. The only significant difference between the two groups was a higher concentration of presepsin in nonsurvivors (*P* = 0.0015). The main clinical characteristics, risk scores (SOFA score at enrollment and SAPS II score), as well as PCT levels, were not significantly different.

**Table 1:** **Baseline clinical characteristics and biomarkers in survivors and nonsurvivors at ICU discharge**^**a**^

**Characteristics**	**Survivors (*n*** **= 50)**	**Decedents (*n*** **= 50)**	***P***
Age (years)	71.6 ± 10.8	71.3 ± 13.6	0.63
Females, *n* (%)	23 (46)	23 (46)	1.00
BMI (kg/m^2^)	25.5 ± 4.9	28.4 ± 7.6	0.09
Randomized to albumin, *n* (%)	27 (54)	27 (54)	1.00
Source of severe sepsis, *n* (%)			
Lungs	19 (38)	19 (38)	1.00
Abdomen	26 (52)	20 (40)	0.23
Urinary tract	8 (16)	10 (20)	0.60
Other	7 (14)	10 (20)	0.42
SAPS II score	50 ± 14	51 ± 12	0.75
SOFA score	8 (6 to 10)	9 (7 to 11)	0.10
Reason for ICU admission, *n* (%)			
Medical	25 (50)	28 (56)	0.55
Emergency surgery	23 (46)	17 (34)	0.22
Elective surgery	2 (4)	5 (10)	0.44
Shock, *n* (%)	40 (80)	34 (68)	0.17
Mechanical ventilation, *n* (%)	44 (88)	45 (90)	0.75
Vasoactive drugs, *n* (%)	37 (74)	36 (72)	0.82
Heart rate (beats/min)	102 ± 27	103 ± 21	0.91
Mean arterial pressure (mmHg)	73 ± 16	73 ± 14	0.78
Central venous pressure (mmHg)	8.0 (6.0 to 13.7)	9.9 (7.3 to 12.5)	0.54
Central venous oxygen saturation (%)	75 (66 to 81)	73 (66 to 80)	0.53
Urine output (ml/h)	40 (16 to 100)	50 (20 to 90)	0.94
Serum lactate (mmol/L)	2.6 (1.6 to 4.2)	2.5 (1.8 to 4.2)	0.67
Plasma presepsin on day 1 (pg/ml)	1,184 (875 to 2,113)	2,269 (1,171 to 4,300)	0.0015
Plasma procalcitonin on day 1 (μg/L)	10.8 (2.7 to 41.9)	18.5 (3.4 to 45.2)	0.31

^a^BMI, Body mass index; SAPS II, Simplified Acute Physiology Score II; SOFA, Sequential Organ Failure Assessment. Continuous variables are presented as mean ± SD or median and interquartile range when not normally distributed. Categorical variables are presented as number (%).

**Figure 1:**
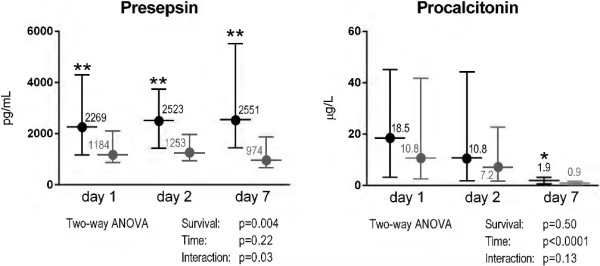
**Time course of plasma concentrations of presepsin and procalcitonin during ICU stay by survival status.** Plasma concentrations of presepsin and procalcitonin 1, 2 and 7 days after enrollment in decedents (*n* = 50, black) and survivors (*n* = 50, gray) at ICU discharge. Data are shown as median and interquartile range. Two-way ANOVA for repeated measurements was done on log-transformed biomarker concentrations. **P < 0.005, *P = 0.01 by Mann–Whitney *U* test.

The evolution of presepsin levels over time in survivors was significantly different from that in nonsurvivors (Figure 1) (*P* value for time–survival interaction = 0.03). At all the three time points, presepsin levels were significantly higher in the decedents than in the survivors (*P* < 0.005). There was no significant difference in presepsin concentrations measured on day 2 or day 7 compared to that on day 1 in both nonsurvivors and survivors. Conversely, PCT levels fell rapidly and similarly in survivors and nonsurvivors (Figure 1), and concentrations were significantly different between the two groups only on day 7 (*P* = 0.01). PCT levels measured on day 2 or day 7 were significantly lower than those on day 1 (*P* < 0.0001 for survivors and *P* < 0.001 for nonsurvivors). The interaction between 7-day time course and survival was significant for SOFA score (*P* = 0.008), but not for serum lactate concentration (*P* = 0.08) or mixed venous oxygen saturation (SvO_2_) (*P* = 0.49).

Plasma concentrations of presepsin were similar between patients with severe sepsis and those with septic shock at the time of study enrollment, both on day 1 (1,571 pg/ml (793 to 2,440) (*n* = 26) vs. 1,485 pg/ml (960 to 3,501) (*n* = 74); *P* = 0.51) and at subsequent times (data not shown). The same was true for SOFA score and PCT (data not shown).

**Figure 2:**
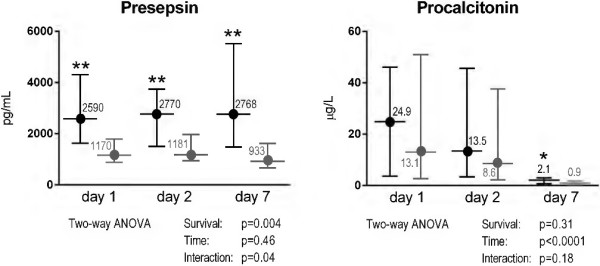
**Time course of plasma concentrations of presepsin and procalcitonin in patients with septic shock during ICU stay by survival status.** Plasma concentrations of presepsin and procalcitonin 1, 2 or 7 days after enrollment in decedents (*n* = 34, black) and survivors (*n* = 40, gray) with septic shock at ICU discharge. Data are shown as median and interquartile range. Two-way ANOVA for repeated measurements was done on log-transformed biomarker concentrations. **P < 0.001, *P = 0.007 by Mann–Whitney *U* test.

In the 74 patients with septic shock, presepsin was significantly higher in decedents than in survivors, both at baseline (2,590 pg/ml (1,631 to 4,310) vs. 1,170 pg/ml (890 to 1,799); *P* = 0.0007) (Figure 2) and on the following days (*P* < 0.001). In contrast, early PCT levels (days 1 and 2) were not different between the two groups with septic shock.

Presepsin levels did not differ significantly in relation to the type of infection by either infection sites or blood cultures (bacterial, fungal, mixed or undetermined) or by the type of bacterial infection (purely Gram-negative, purely Gram-positive, mixed or undetermined; data not shown).

Patients were stratified according to the time from fulfillment of inclusion criteria and study enrollment (within 6 hours or between 6 and 24 hours). In patients with early enrollment (within 6 hours), plasma concentrations of presepsin on day 1 were not significantly different between decedents (2,138 pg/ml (1,062 to 3,101); *n* = 24) and survivors at ICU discharge (1,335 pg/ml (879 to 2,856) (*n* = 25); *P* = 0.17). The same was true for PCT (23.2 μg/L (3.3 to 64.1) vs. 10.9 μg/L (2.8 to 30.8); *P* = 0.20). However, the plasma concentration of presepsin on day 1 was significantly higher in decedents randomized between 6 and 24 hours after the onset of inclusion criteria (2,621 pg/ml (1,223 to 4,959); *n* = 26) than in the survivors at ICU discharge (1,044 pg/ml (875 to 1,331) (*n* = 25); *P* = 0.003), whereas PCT did not differ (16.2 μg/L (3.4 to 40.6) vs. 7.1 μg/L (2.7 to 41.8); *P* = 0.88).

### Plasma presepsin concentration in relation to mortality

The prognostic value of presepsin was evaluated at ICU discharge and during follow-up period on days 28 and 90 after study enrollment according to prespecified study endpoints. In univariate Cox proportional hazards models (Table 2), presepsin on day 1 was associated with mortality at ICU discharge (HR = 1.65 (95% CI = 1.22 to 2.24) for each 1 unit increase in log-transformed concentration; *P* = 0.0012), at 28 days (HR = 1.73 (95% CI = 1.32 to 2.27); *P* < 0.001) and at 90 days (HR = 1.50 (95% CI = 1.18 to 1.91); *P* = 0.001). The corresponding HRs and 95% CIs for PCT were 1.01 (95% CI =0.87 to 1.18; *P* =0.88), 1.07 (95% CI = 0.92 to 1.25; *P* =0.40), and 1.03 (95% CI =0.91 to 1.18; *P* =0.63). After adjustment for clinically relevant variables, including SAPS II score, SvO_2_ level and mean arterial pressure 6 hours after study enrollment, as well as SOFA score and serum lactate level on day 1, presepsin on day 1 remained independently related to outcome at ICU discharge (HR = 1.48 (95% CI = 1.04 to 2.11; *P* = 0.03) and after 28 days (HR = 1.54 (95% CI = 1.12 to 2.12); *P* = 0.01) (Table 2), but not after 90 days (HR = 1.29 (95% CI = 0.96 to 1.72); *P* = 0.09).

**Table 2:** **Univariate and multivariate Cox models for mortality**^**a**^

**Time**	**Biomarker**	**ICU mortality**				**28-day mortality**				**90-day mortality**			
		**Univariate**		**Multivariate**		**Univariate**		**Multivariate**		**Univariate**		**Multivariate**	
Day 1	Presepsin	1.65 (1.22 to 2.24)	0.001	1.51 (1.05 to 2.17)	0.03	1.73 (1.32 to 2.27)	<0.0001	1.55 (1.12 to 2.13)	0.008	1.50 (1.18 to 1.92)	0.001	1.28 (0.96 to 1.71)	0.09
	Procalcitonin	1.01 (0.87 to 1.18)	0.88	0.89 (0.75 to 1.05)	0.15	1.07 (0.92 to 1.25)	0.40	0.97 (0.84 to 1.12)	0.68	1.03 (0.91 to 1.18)	0.63	0.94 (0.83 to 1.06)	0.32
Day 2	Presepsin	1.84 (1.33 to 2.54)	0.0002	1.83 (1.19 to 2.81)	0.006	2.02 (1.49 to 2.75)	<0.0001	1.93 (1.27 to 2.92)	0.002	1.73 (1.31 to 2.28)	0.0001	1.47 (1.02 to 2.12)	0.04
	Procalcitonin	1.00 (0.86 to 1.16)	0.97	0.82 (0.68 to 0.98)	0.03	1.05 (0.90 to 1.23)	0.54	0.85 (0.70 to 1.02)	0.08	1.06 (0.92 to 1.21)	0.43	0.86 (0.73 to 1.02)	0.08
Day 7	Presepsin	1.77 (1.30 to 2.41)	0.0003	1.83 (1.22 to 2.74)	0.004	2.11 (1.55 to 2.89)	<0.0001	2.13 (1.41 to 3.21)	0.0003	1.84 (1.41 to 2.41)	<0.0001	1.75 (1.23 to 2.48)	0.002
	Procalcitonin	1.12 (0.93 to 1.33)	0.23	0.97 (0.78 to 1.21)	0.78	1.29 (1.06 to 1.57)	0.01	1.11 (0.87 to 1.42)	0.39	1.25 (1.05 to 1.47)	0.01	1.08 (0.88 to 1.33)	0.46

^a^Risk is presented as hazard ratio and 95% confidence interval with an increment of 1 unit after logarithmic transformation of the biomarker concentration. The following covariates, deemed important clinical variables, were considered in the multivariable models: Simplified Acute Physiology Score II score, Sequential Organ Failure Assessment score, serum lactate concentration, mean arterial pressure, central venous oxygen saturation and randomized treatment (albumin vs. crystalloids).

Presepsin concentrations, but not PCT concentrations, on days 2 and 7 were independently related to mortality in the ICU, as well as on days 28 and 90 (Table 2). The SOFA score was the only clinical variables independently associated with mortality in the multivariable models including PCT; when presepsin was included in place of PCT in these models, the SOFA score was no longer significantly associated with mortality (data not shown).

### Prognostic accuracy of presepsin

The prognostic accuracy of presepsin was evaluated on the basis of ROC curves, yielding AUCs for ICU survival of 0.69, 0.70 and 0.74 on days 1, 2 and 7, respectively (Table 3). The SOFA score had similar accuracy. Corresponding values for PCT were 0.56, 0.55 and 0.64, respectively.

**Table 3:** **Prognostic accuracy of presepsin, procalcitonin and clinical risk scores**^**a**^

**ICU survival**									**28 *- *** **day survival**								**90 *- *** **day survival**							
	**AUC (95% CI)**	**Optimal cutoff**	**Sensitivity (%)**	**Specificity (%)**	**PPV (%)**	**NPV (%)**	**LR+**	**LR-**	**AUC (95% CI)**	**Optimal cutoff**	**Sensitivity (%)**	**Specificity (%)**	**PPV (%)**	**NPV (%)**	**LR+**	**LR-**	**AUC (95% CI)**	**Optimal cutoff**	**Sensitivity (%)**	**Specificity (%)**	**PPV (%)**	**NPV (%)**	**LR+**	**LR-**
Presepsin																								
Day 1	0.69 (0.58 to 0.79)	1631	66.7	74.0	71	70	2.56	0.45	0.72 (0.61 to 0.82)	1631	67.4	70.9	64	74	2.32	0.46	0.66 (0.55 to 0.77)	1631	62.5	71.4	76	58	2.19	0.53
Day 2	0.70 (0.59 to 0.87)	1718	69.4	73.5	72	71	2.62	0.42	0.74 (0.64 to 0.85)	1718	74.4	72.7	68	78	2.73	0.35	0.68 (0.57 to 0.79)	1407	77.2	61.0	73	66	1.98	0.37
Day 7	0.74 (0.64 to 0.84)	1606	72.0	70.0	71	71	2.40	0.40	0.74 (0.64 to 0.84)	2028	63.6	76.8	68	73	2.74	0.47	0.70 (0.60 to 0.81)	1453	70.7	64.3	73	61	1.98	0.46
Procalcitonin																								
Day 1	0.56 (0.44 to 0.68)	14.27	60.4	58.0	58	60	1.44	0.68	0.55 (0.44 to 0.67)	14.27	60.5	56.4	52	65	1.39	0.70	0.53 (0.41 to 0.65)	14.27	57.1	57.1	64	50	1.33	0.75
Day 2	0.55 (0.44 to 0.67)	8.88	60.4	55.1	57	59	1.35	0.72	0.53 (0.41 to 0.65)	8.88	59.5	52.7	49	63	1.26	0.77	0.55 (0.43 to 0.67)	8.88	60.7	58.5	67	52	1.46	0.67
Day 7	0.64 (0.54 to 0.75)	1.51	56.0	74.0	68	63	2.15	0.59	0.65 (0.54 to 0.76)	1.47	61.4	73.2	64	71	2.29	0.53	0.63 (0.52 to 0.74)	1.47	55.2	76.2	76	55	2.32	0.59
SOFA score																								
Day 1	0.69 (0.59 to 0.80)	9	65.3	68.8	68	66	2.09	0.50	0.68 (0.58 to 0.79)	9	67.4	66.7	62	72	2.02	0.49	0.68 (0.57 to 0.79)	9	61.4	70.0	74	57	2.05	0.55
Day 2	0.67 (0.56 to 0.78)	8	73.9	54.2	61	68	1.61	0.48	0.72 (0.61 to 0.82)	9	70.0	64.8	60	74	1.99	0.46	0.64 (0.53 to 0.76)	8	70.4	55.0	68	58	1.56	0.54
Day 7	0.75 (0.65 to 0.85)	7	59.6	83.0	78	67	3.50	0.49	0.75 (0.65 to 0.85)	7	61.0	79.2	69	72	2.94	0.49	0.71 (0.60 to 0.81)	6	66.7	65.0	72	59	1.91	0.51
SAPS II																								
Day 1	0.51 (0.39 to 0.62)	49	56.0	48.0	52	52	1.08	0.92	0.63 (0.52 to 0.74)	51	61.4	60.7	55	67	1.56	0.64	0.58 (0.47 to 0.70)	49	60.3	54.8	65	50	1.33	0.72

^a^95% CI, 95% confidence interval; LR+, positive likelihood ratio; LR-, negative likelihood ratio; NPV, negative predictive value; PPV, positive predictive value; Simplified Acute Physiology Score II score, Sequential Organ Failure Assessment score. The areas under the receiver operating characteristic curve (AUCs) are shown as means (95% confidence intervals).

## Discussion

Severe sepsis is still a major challenge in critical care. Over the past ten years, many efforts have improved several aspects of its treatment and prognosis, such as earlier identification of the potential infection, early etiologic therapy and early implementation of adequate supportive therapy while avoiding potential harmful effects [1,16]. Nonetheless, the early stratification of patients with severe sepsis and their prognosis, as well as accurate monitoring of the effects of clinical treatment, is still an unsolved issue. Clinical scores have been introduced in clinical practice to partially satisfy this task, such as the SOFA score [10] and the Acute Physiology and Chronic Health Evaluation II score [17], but the state of the art in diagnosing and monitoring severe sepsis on the basis of circulating biomarkers relies on PCT at the onset and during the course of the disease [18]. Despite all the data available and widespread clinical implementation, PCT has shown limited value for risk stratification and prognostication.

The 55 kDa glycoprotein CD14 is expressed on the membrane of monocytes and macrophages. It facilitates the Toll-like receptor 4–specific inflammatory reaction whereby soluble CD14 (sCD14) is released into the circulation and serves as a mediator of the response to LPS from infectious agents. Simultaneously, a 13 kDa fragment of sCD14, named sCD14-ST or presepsin, is formed [19]. Although its biological function remains unclear, it appears to be released in the plasma as a consequence of cellular phagocytosis after bacterial infection and is therefore an indirect marker of sepsis [20]. It was identified less than one decade ago in patients with sepsis and appears to be superior to other biomarkers (IL-6 and PCT), as well as to infection site and blood culture, for the diagnosis of severe sepsis of bacterial origin [7,20].

In our present multicenter, case–control study, we investigated the potential prognostic power of presepsin during ICU stay in patients with severe sepsis. A recent study of 106 patients presenting at the emergency departments of two hospital centers with suspected sepsis or septic shock showed that presepsin measured on the first medical evaluation (but not successively) was associated with 60-day mortality by univariate analysis [21]. In our study, presepsin levels on day 1 remained independently associated with mortality at early stages (at ICU discharge and after 28 days), even after correction for the individual clinical variables considered to be the most important parameters of the resuscitation phase (mean arterial pressure, serum lactate level and central venous oxygen saturation) [1,11]. The models were also adjusted for widely used clinical scores of overall severity (such as SAPS II score) [22] or multiorgan failure (such as SOFA score) [10]. No independent association was observed between any of these variables and mortality when presepsin was included in the models. Taken together, these findings suggest that presepsin is a robust circulating biomarker for early stratification of the severity of sepsis, as well as for patient prognosis. Although there is contrasting evidence on the independent prognostic value of PCT in septic patients [23,24], there is a consensus that its main clinical use is to aid in the monitoring of antibiotic therapy. Sepsis is a complex syndrome with an initial phase (usually the first 48 hours) in which appropriate etiological therapy and adequate supportive treatment must be established, followed by a second phase when multiple organ failures may occur. Presepsin, being superior as an early prognostic factor to clinical parameters in both phases, may have unique characteristics encompassing the complexity of the whole syndrome.

With regard to the time course of presepsin, we observed a significant difference between survivors and nonsurvivors. PCT decreased in both groups, as already observed by others [25]. This suggests that presepsin may also be used for monitoring the efficacy of the therapy adopted—etiologic, supportive or both—as previously suggested [6,20]. Although our study design did not allow us to demonstrate any direct association between the treatment applied and late levels of presepsin, we speculate that the drop in presepsin levels in patients who survived indicates a beneficial effect of the treatment by reducing the activity of the bacterial infection or by controlling its systemic reactivity. Because presepsin has previously been identified as a promising biomarker for the diagnosis of sepsis, superior even to conventional markers and blood culture [6,7], it is conceivable that early assessment of circulating presepsin might help in making early, correct diagnoses, as well as in monitoring the appropriateness of the therapy implemented.

When we compared presepsin levels to clinical scores, the prognostic accuracy of presepsin level appeared to be similar to that of the SOFA score for both early (ICU discharge and after 28 days) and late mortality (90-day). This seems reasonable because the SOFA score—the most commonly used clinical score to assess the development of organ failures—relies on the concept that the higher the severity and number of organ failures during the course of sepsis, the greater the likelihood of death [10]. It is tempting to speculate, too, that the greater the host’s biological response to the microorganism (due either to its virulence or to the immune reaction) and therefore, indirectly, the higher the levels of plasma presepsin, the greater the severity of sepsis, the risk of multiple organ dysfunction and ultimately the risk of death.

Some limitations of the present study deserve consideration. First, the relatively small sample size did not allow in-depth analysis of the relationships between presepsin levels and disease characteristics (for example, infecting organism, source of sepsis) or severity (for example, physiological score, multiorgan failure) as well as the therapy applied. Second, we conducted a retrospective case–control study with inherent selection bias and thus we might have overlooked some confounding factors. Although we cannot exclude the possibility that selecting the study population according to early ICU survival might have partially affected our findings, the evaluation of outcomes even after 28 and 90 days likely supported the plausibility of our observations, which await further validation. Third, although extensive daily monitoring of circulating biomarkers is important during the early management of sepsis [26], only three measurements (on days 1, 2 and 7) were available.

## Conclusion

In this study, we show for the first time that presepsin is an early marker of mortality with better prognostic performance than PCT and that it may be proposed as an aid in risk stratification strategies in the septic patient. These preliminary findings provide a solid basis for future, more extensive evaluation of presepsin as a biomarker for severe sepsis. More insight is needed regarding the pathophysiological conditions associated with presepsin release, both in experimental models of sepsis and in well-characterized patients. The added value of this biomarker for clinical decision-making in terms of diagnosis, risk stratification and therapy monitoring should also be delineated. The clinical indications for presepsin should be confirmed and validated in large-scale, independent cohorts of unselected patients with severe sepsis or septic shock.

## Key messages

• Circulating presepsin (sCD14-ST) levels have a significantly different trajectory over the ICU stay in decedents with severe sepsis and septic shock compared to survivors.

• Presepsin is an early marker of mortality, independently of clinical risk factors, with better prognostic performance than PCT.

## Abbreviations

ALBIOS: Albumin Italian Outcome Sepsis; AUC: Area under the curve; LPS: Lipopolysaccharide; PCT: Procalcitonin; ROC: Receiver operating characteristic; SAPS II: Simplified Acute Physiology Score II; sCD14-ST: Soluble N-terminal fragment of cluster of differentiation marker protein 14; SOFA: Sequential Organ Failure Assessment; SvO2: Mixed venous oxygen saturation.

## Competing interests

SM, PC, RL and LG received a limited research support grant from Mitsubishi Chemical Europe GmbH, the manufacturer of the presepsin assay. RT is an employee at Mitsubishi Chemical Europe GmbH. Diagnostics Engineering & Research GmbH (DIAneering GmbH) performed the laboratory measurements and served as consultants to Mitsubishi Chemical Europe GmbH.

## Authors’ contributions

SM and PC were involved in the acquisition of data, data processing, study design, statistical analysis and manuscript writing and drafting. ES, RT, MP, GS, RF, TM and SI participated in the acquisition and interpretation of data and in the final revision of the manuscript. CF was involved in data processing, performed the statistical analysis and participated in the writing and drafting of the manuscript. RL was involved in the acquisition of data, the study design and the final revision of the manuscript. MR, GT and LG were involved in the study design and the final revision of the manuscript. All authors read and approved the final manuscript.

## Supplementary Material

Additional file 1 **ALBIOS Biomarkers Substudy: Participating centers.** List of participating centers. Click here for file

Additional file 2 **ALBIOS Biomarkers Substudy: Participating centers and ethical bodies. **List of participating centers and their ethical bodies. Click here for file

Additional file 3 **Baseline clinical characteristics according to median presepsin concentration at study entry.** Clinical characteristics at baseline are compared in patients with plasma presepsin concentration less than or greater than or equal to 1,494 pg/ml. Click here for file
