# Supplementary material for: Presepsin (soluble CD14 subtype) and procalcitonin levels for mortality prediction in sepsis: data from the Albumin Italian Outcome Sepsis trial
Source: Crit Care. 2014 Jan 7;18(1):R6. doi: 10.1186/cc13183 (PMC4056046; doi:10.1186/cc13183)
Supplement: Additional file 1 — ALBIOS Biomarkers Substudy: Participating centers. List of participating centers. [file cc13183-S1.docx]

**Additional file 1**

**ALBIOS Biomarkers Substudy - Participating centers and investigators**

*Milano - Fondazione IRCCS Ca' Granda - Ospedale Maggiore Policlinico* (Paola Bruzzone, Francesca Pagan, Riccarda Russo); *Monza – Ospedale San Gerardo* (Andrea Confalonieri, Chiara Abbruzzese, Beatrice Vergnano)*; Bologna - Policlinico Universitario S. Orsola Malpighi* (Stefano Faenza, Antonio Siniscalchi, Elisabetta Pierucci); *Milano - A.O. San Paolo-Polo Universitario* (Andrea Noto, Angelo Pezzi, Paolo Spanu); *Borgo San Lorenzo - Ospedale del Mugello* (Vieri Parrini, Roberto Oggioni); *Orbetello Scalo - Ospedale S. Giovanni di Dio* (Giovanni Stefano Pasetti, Maria Cinzia Casadio, Rosa Buontempo); *Rho – Ospedale di Circolo* (Sara Carrer, Francesca Piccoli, Tatiana Rizzi); *Roma - Università Cattolica - Policlinico Universitario A. Gemelli* (Anselmo Caricato, Monica La Sala, Alessandra Antonaci); *Legnano – Ospedale Civile* (Paola Fassini, Silvia Paganini, Virginia Porta); *Sesto San Giovanni - Ospedale "Città di Sesto San Giovanni"* (Gabriella Moise, Silvia Marelli, Mirella Furia); *Grosseto - Ospedale della Misericordia* (Maria Cristina Urbano, Roberta Carobbi, Simona Poleni); *San Donato Milanese - IRCCS Policlinico San Donato* (Hassan Kandil, Andrea Ballotta, Fabrizio Bettini); *Riccione - Ospedale Ceccarini* (Manlio Sanseverino, Alessandro Gatta, Francesca Cecchini); *Desio – Ospedale di Desio* (Luca Guatteri, Gabriella Ciceri); *Milano – A.O. Luigi Sacco* (Ferdinando Raimondi, Roberto Colombo); *Treviglio – Ospedale di Treviglio* (Sandra Ferraris, Massimo Borelli); *Rozzano – Istituto Clinico Humanitas* (Valentina Bellato, Franco Cancellieri); *Seriate – Azienda Bolognini* (Silvia Senni, Ester Bertocchi); *Crema – Ospedale Maggiore* (Paola Ferri, Gianpietro Moioli); *Genova - Azienda Ospedaliera Universitaria "San Martino"* (Andrea Fedele, Alexandra Molin); *Reggio Emilia - Arcispedale S. Maria Nuova* (Giovanni Salati, Pierpaolo Salsi); *Macerata - Ospedale Macerata ASUR Marche* (Emanuela Brunori, Daniele Elisei); *Pavia - IRCCS Fondazione Policlinico” San Matteo” / Rianimazione 1* (Giuseppe Maggio, Federico Guardia Nicola); *Aosta - Azienda USL “V. Parini” Valle d'Aosta* (Marco Cavana, Giacomo Morelli); *Sampierdarena - Azienda Ospedaliera "Villa Scassi"* (Arturo Guarino, Michele Isetta); *Firenze - Ospedale San Giovanni di Dio* (Giorgio Tulli, Valerio Mangani); *Milano - Ospedale San Giuseppe - Milano Cuore* (Nicola Rossi); *Pavia - IRCCS Fondazione Policlinico "San Matteo" / Rianimazione 2* (Marta Ferrari); *Candiolo - Istituto per la Cura e la Ricerca del Cancro* (Francesco Bona); *Torino - ASO Universitaria S. Giovanni Battista di Torino – Molinette* (Monica Vay); *Bagno a Ripoli - Azienda Sanitaria Santa Maria Annunziata* (Teresa Bartoli); *Torino - A.O. Ordine Mauriziano, Presidio Ospedaliero "Umberto I"* (Mauro Gallo); *Manerbio – Ospedale di Manerbio* (Katiuscia Vettoretto); *Sondalo - Azienda Ospedaliera della Valtellina e della Valchiavenna* (Mauro Della Morte); *Garbagnate - Azienda Ospedaliera "Guido Salvini"* (Enrico Boselli); *Lecce - Ospedale "Vito Fazzi"* (Daniela Puscio); *Camposampiero - ULSS 15 Alta Padovana* (Monia Bovo); *Perugia – Ospedale Santa Maria della Misericordia* (Antonio Galzerano); *Pistoia - Ospedale del Ceppo* (Manuela Carli); *Palermo - Azienda Ospedaliera “V. Cervello”* (Giovanni Zagara).
