# Supplementary material for: Presepsin (soluble CD14 subtype) and procalcitonin levels for mortality prediction in sepsis: data from the Albumin Italian Outcome Sepsis trial
Source: Crit Care. 2014 Jan 7;18(1):R6. doi: 10.1186/cc13183 (PMC4056046; doi:10.1186/cc13183)
Supplement: Additional file 2 — ALBIOS Biomarkers Substudy: Participating centers and ethical bodies. List of participating centers and their ethical bodies. [file cc13183-S2.docx]

**Additional file 2**

**ALBIOS Biomarkers Substudy - Participating centers and ethical bodies**

*Milano - Fondazione IRCCS Ca' Granda - Ospedale Maggiore Policlinico* (Lead ethical committee, Comitato Etico della Fondazione IRCCS Ca’ Granda – Ospedale Maggiore Policlinico di Milano); *Monza – Ospedale San Gerardo* (Comitato Etico dell'Azienda Ospedaliera S. Gerardo di Monza)*; Bologna - Policlinico Universitario S. Orsola Malpighi* (Comitato Etico Indipendente dell'Azienda Ospedaliero- Universitaria Policlinico S. Orsola-Malpighi di Bologna); *Milano - A.O. San Paolo-Polo Universitario* (Comitato Etico dell'Azienda Ospedaliera San Paolo - Milano); *Borgo San Lorenzo - Ospedale del Mugello* (Comitato Etico per la Sperimentazione Clinica dei Medicinali dell'Azienda Sanitaria di Firenze); *Orbetello Scalo - Ospedale S. Giovanni di Dio* (Comitato Etico per la Sperimentazione dei Farmaci della AUSL 9 di Grosseto); *Rho – Ospedale di Circolo* (Comitato Etico dell’Azienda Ospedaliera "Guido Salvini" di Garbagnate Milanese); *Roma - Università Cattolica - Policlinico Universitario A. Gemelli* (Comitato Etico dell’Università Cattolica del Sacro Cuore di Roma - Facoltà di Medicina e Chirurgia Agostino Gemelli e Policlinico Universitario); *Legnano – Ospedale Civile* (Comitato Etico dell'Azienda Ospedaliera Ospedale Civile di Legnano); *Sesto San Giovanni - Ospedale "Città di Sesto San Giovanni"* (Comitato Etico dell’Azienda Ospedaliera Istituti Clinici di Perfezionamento di Milano); *Grosseto - Ospedale della Misericordia* (Comitato Etico per la Sperimentazione dei Farmaci della AUSL 9 di Grosseto); *San Donato Milanese - IRCCS Policlinico San Donato* (Comitato Etico Indipendente della ASL della provincia di Milano 2 di Melegnano); *Riccione - Ospedale Ceccarini* (Comitato Etico di Area Vasta Romagna di Cesena e Istituto Scientifico Romagnolo per lo Studio e la Cura dei Tumori di Meldola); *Desio – Ospedale di Desio* (Comitato Etico dell’Azienda Ospedaliera di Vimercate); *Milano – A.O. Luigi Sacco* (Comitato Etico Locale per la Sperimentazione Clinica dell'Ospedale Luigi Sacco di Milano); *Treviglio – Ospedale di Treviglio* (Comitato Etico dell'Azienda Ospedaliera di Treviglio); *Rozzano – Istituto Clinico Humanitas* (Comitato Etico Indipendente della ASL della provincia di Milano 2 di Melegnano); *Seriate – Azienda Bolognini* (Comitato Etico Indipendente dell'Azienda Ospedaliera Bolognini di Seriate); *Crema – Ospedale Maggiore* (Comitato Etico dell'Azienda Ospedaliera Ospedale Maggiore di Crema); *Genova - Azienda Ospedaliera Universitaria "San Martino"* (Comitato Etico dell’Azienda Ospedaliera Universitaria S. Martino di Genova); *Reggio Emilia - Arcispedale S. Maria Nuova* (Comitato Etico Provinciale di Reggio Emilia); *Macerata - Ospedale Macerata ASUR Marche* (Comitato Etico Internazionale della A.S.U.R .Zona Territoriale 8 di Civitanova Marche e Zona Territoriale 9 di Macerata); *Pavia - IRCCS Fondazione Policlinico” San Matteo” / Rianimazione 1* (Comitato di Bioetica della Fondazione IRCCS Policlinico S. Matteo di Pavia); *Aosta - Azienda USL “V. Parini” Valle d'Aosta* (Comitato Etico della AUSL della Valle d’Aosta di Aosta); *Sampierdarena - Azienda Ospedaliera "Villa Scassi"* (Comitato Etico della ASL 3 Genovese di Genova); *Firenze - Ospedale San Giovanni di Dio* (Comitato Etico per la Sperimentazione Clinica dei Medicinali dell'Azienda Sanitaria di Firenze); *Milano - Ospedale San Giuseppe - Milano Cuore* (Comitato Etico indipendente dell'Ospedale San Giuseppe - Ordine Ospedaliero di San Giovanni di Dio - Fatebenefratelli di Milano); *Pavia - IRCCS Fondazione Policlinico "San Matteo" / Rianimazione 2* (Comitato di Bioetica della Fondazione IRCCS Policlinico S. Matteo di Pavia); *Candiolo - Istituto per la Cura e la Ricerca del Cancro* (Comitato Etico Interaziendale delle ASO OIRM/S. Anna e Ordine Mauriziano di Torino); *Torino - ASO Universitaria S. Giovanni Battista di Torino – Molinette* (Comitato Etico Interaziendale delle ASO S. Giovanni Battista e CTO/CRF/Maria Adelaide di Torino); *Bagno a Ripoli - Azienda Sanitaria Santa Maria Annunziata* (Comitato Etico per la Sperimentazione Clinica dei Medicinali dell'Azienda Sanitaria di Firenze); *Torino - A.O. Ordine Mauriziano, Presidio Ospedaliero "Umberto I"* (Comitato Etico Interaziendale delle ASO OIRM/S. Anna e Ordine Mauriziano di Torino); *Manerbio – Ospedale di Manerbio* (Comitato Etico dell'Azienda Ospedaliera di Desenzano del Garda); *Sondalo - Azienda Ospedaliera della Valtellina e della Valchiavenna* (Comitato Etico dell'Azienda Ospedaliera della Valtellina e della Valchiavenna); *Garbagnate - Azienda Ospedaliera "Guido Salvini"* (Comitato Etico dell’Azienda Ospedaliera "Guido Salvini" di Garbagnate Milanese); *Lecce - Ospedale "Vito Fazzi"* (Comitato Etico dell'Azienda ASL di Lecce); *Camposampiero - ULSS 15 Alta Padovana* (Comitato Etico per la Sperimentazione Clinica della provincia di Padova); *Perugia – Ospedale Santa Maria della Misericordia* (Comitato Etico delle Aziende Sanitarie dell´Umbria di Perugia); *Pistoia - Ospedale del Ceppo* (Comitato Etico per la Sperimentazione Clinica dei Medicinali della AUSL 3 di Pistoia); *Palermo - Azienda Ospedaliera “V. Cervello”* (Comitato Etico dell’Azienda Ospedaliera Vincenzo Cervello di Palermo).
