# Supplementary material for: Presepsin (soluble CD14 subtype) and procalcitonin levels for mortality prediction in sepsis: data from the Albumin Italian Outcome Sepsis trial
Source: Crit Care. 2014 Jan 7;18(1):R6. doi: 10.1186/cc13183 (PMC4056046; doi:10.1186/cc13183)
Supplement: Additional file 3 — Baseline clinical characteristics according to median presepsin concentration at study entry. Clinical characteristics at baseline are compared in patients with plasma presepsin concentration less than or greater than or equal to 1,494 pg/ml. [file cc13183-S3.docx]

**Additional file 3: Baseline clinical characteristics according to median presepsin concentration at study entry**

| **Characteristics** | **Presepsin < 1494 pg/mL** (n= 49) | **Presepsin ≥ 1494 pg/mL** (n= 49) | **P** |
| --- | --- | --- | --- |
| Age (years) | 72.7±11.1 | 70.9±12.7 | 0.52 |
| Females (no. (%)) | 24 (49) | 20 (41) | 0.42 |
| BMI (kg/m^2^) | 26.4±5.7 | 27.3±7.3 | 0.69 |
| Randomized allocation to albumin (no. (%)) | 27 (55) | 26 (53) | 0.84 |
|  |  |  |  |
| ***Source of severe sepsis*** *(no. (%))* |  |  |  |
| Lungs | 22 (45) | 14 (29) | 0.09 |
| Abdomen | 24 (49) | 22 (45) | 0.69 |
| Urinary tract | 8 (16) | 9 (18) | 0.79 |
| Other | 6 (12) | 11 (22) | 0.18 |
| SAPS II | 49±11 | 52±14 | 0.34 |
| SOFA score | 7 [6-9] | 9 [7-11] | 0.002 |
| ***Reason for ICU admission*** *(no. (%))* |  |  |  |
| Medical | 25 (51) | 26 (53) | 0.84 |
| Emergency surgery | 20 (41) | 20 (41) | 1.00 |
| Elective surgery | 4 (8) | 3 (6) | 1.00 |
| Shock (no. (%)) | 37 (76) | 36 (73) | 0.82 |
| Mechanical ventilation (no. (%)) | 46 (94) | 41 (84) | 0.11 |
| Vasoactive drugs (no. (%)) | 37 (76) | 35 (71) | 0.65 |
| Heart rate (beats/min) | 101±25 | 104±23 | 0.65 |
| Mean arterial pressure (mmHg) | 75±15 | 70±14 | 0.15 |
| Central venous pressure (mmHg) | 9 [6.9-12.5] | 10 [7-13.7] | 0.48 |
| Central venous oxygen saturation (%) | 74 [66-81] | 73 [66-79] | 0.62 |
| Urine output (mL/hr) | 80 [30-105] | 30 [0-60] | 0.0005 |
| Serum lactate (mmol/L) | 2.1 [1.7-4.1] | 2.9 [1.9-4.0] | 0.13 |
| Plasma procalcitonin on day 1 (μg/L) | 14.23 [1.7-40.6] | 15.5 [3.4-60.2] | 0.26 |

Continuous variables are presented as mean ±SD or median and interquartile range when not normally distributed; categorical variables as number (%). Abbreviations: ICU = intensive care unit, SAPS II = Simplified Acute Physiology Score II; SOFA = Sequential Organ Failure Assessment score.
